# Editorial: Infections in the intensive care unit, volume III

**DOI:** 10.3389/fmed.2025.1719505

**Published:** 2025-10-30

**Authors:** Cheng Zhu, Bin Lin, Zhongheng Zhang, Yuetian Yu

**Affiliations:** ^1^Department of Disease Prevention and Control, Ruijin Hospital, Shanghai Jiao Tong University, School of Medicine, Shanghai, China; ^2^Key Laboratory of Intelligent Pharmacy and Individualized Therapy of Huzhou, Huzhou, China; ^3^Department of Pharmacy, Changxing People's Hospital, Changxing Branch, Second Affiliated Hospital of Zhejiang University School of Medicine, Huzhou, China; ^4^Department of Emergency Medicine, Sir Run-Run Shaw Hospital, Zhejiang University School of Medicine, Hangzhou, China; ^5^Provincial Key Laboratory of Precise Diagnosis and Treatment of Abdominal Infection, Sir Run Run Shaw Hospital, Zhejiang University School of Medicine, Hangzhou, China; ^6^Department of Critical Care Medicine, Renji Hospital, School of Medicine, Shanghai Jiao Tong University, Shanghai, China

**Keywords:** infection, intensive care unit, sepsis, antibiotic stewardship, therapeutic drug monitoring, subphenotypes

Severe infections and sepsis remain the most critical challenges in the field of intensive care medicine. Given their high incidence and mortality rates, they have garnered widespread global attention. The “5 Facts and 5 Actions” initiative launched on World Sepsis Day 2025 once again emphasized the need to further enhance awareness of sepsis and mobilize all medical resources to improve patient outcomes. Since 2023, our team has been preparing a Research Topic on “Infections in the ICU,” aiming to share advancements in both clinical and basic research in this field, with the goal of transforming clinical perspectives and translating these insights into actionable practices that can improve patient prognosis (1). We are deeply honored to have completed the compilation of the third volume with your support and assistance. Ultimately, we selected 22 out of nearly 70 submissions for publication.

This collection of 22 articles represents the cutting-edge research and clinical insights from experts worldwide, addressing the most pressing challenges in the management of critically ill patients with infections. The contributions herein collectively paint a picture of a field in rapid, transformative evolution, moving from reactive paradigms to proactive, personalized, and precision medicine.

## The unabating challenge: the severity and complexity of ICU infections

The intensive care unit (ICU) remains the epicenter of the most severe and life-threatening infections. The patients within its walls are characterized by a perfect storm of vulnerability: compromised immune defenses due to underlying disease, breaches in natural anatomical barriers from invasive devices (endotracheal tubes, central venous catheters, urinary catheters), and the frequent use of broad-spectrum antibiotics. This environment is a crucible for the selection and proliferation of multidrug-resistant organisms (MDROs) (2).

This Research Topic powerfully underscores the persistent and evolving threat of antimicrobial resistance (AMR). Studies within this Research Topic detail the grim reality of infections caused by carbapenem-resistant *Enterobacteriaceae* (CRE), *Acinetobacter baumannii, Pseudomonas aeruginosa*, and vancomycin-resistant *Enterococci* (VRE). These pathogens are often extensively or pan-drug resistant, leaving clinicians with a dwindling arsenal of therapeutic options. The clinical consequences are starkly illustrated: prolonged mechanical ventilation (MV), extended ICU and hospital lengths of stay, exorbitant healthcare costs, and unacceptably high mortality rates.

A cross-sectional survey in many countries and regions around the world has provided sobering data on the dire prognoses associated with these infections, reinforcing that AMR is not a future threat but a present-day catastrophe in ICUs globally (3). This volume serves as a critical reminder that the foundational battle against ICU infections begins with robust infection prevention and control (IPC) measures and antimicrobial stewardship (AMS), but it must be augmented by technological advancement.

## Revolutionizing diagnostics: the dawn of rapid, precision pathogen identification

A central theme emerging from this Research Topic is the paradigm shift in microbiological diagnostics. The traditional culture-based methods, with their 48- to 72-h turnaround times, are increasingly recognized as inadequate for guiding initial, life-saving therapy in sepsis. The articles in this section herald a new era of rapid diagnostics, which is pivotal for de-escalating empiric broad-spectrum therapy and initiating targeted treatment sooner.

Multiple studies explore the clinical utility of molecular and multiplex polymerase chain reaction (PCR) panels that can identify a plethora of pathogens and key resistance genes (e.g., blaKPC, blaNDM, mecA) directly from blood or respiratory samples within hours. Some research works have demonstrated how the implementation of such technology was associated with a significant reduction in time to effective therapy and a shorter duration of unnecessary anti-MRSA or anti-pseudomonal coverage.

Furthermore, the horizon of diagnostics is expanding toward more comprehensive and unbiased techniques. Metagenomic next-generation sequencing (mNGS) allows for the detection of virtually all nucleic acids in a sample, proving invaluable for diagnosing culture-negative infections, uncovering unexpected or fastidious pathogens, and characterizing complex polymicrobial communities (4, 5). The application of these advanced diagnostics is moving us from a state of educated guesswork to one of precise pathogen identification, forming the bedrock upon which modern antimicrobial stewardship is built.

## Replenishing the arsenal: novel antimicrobial agents and strategies

Confronted with the rise of pan-resistant bacteria, the development of novel antimicrobial agents is not just a research priority but a clinical necessity. This Research Topic features several contributions evaluating new antibiotics and alternative therapeutic strategies. Prominent among these are the newer generations of β-lactam/β-lactamase inhibitor (BLBLI) combinations (e.g., ceftolozane-tazobactam, ceftazidime-avibactam, meropenem-vaborbactam). Some studies have provided real-world evidence on the efficacy and safety of these agents for treating infections caused by extended-spectrum β-lactamase (ESBL)-producing and carbapenem-resistant gram-negative bacteria. These drugs represent a critical step forward, yet the rapid emergence of resistance to even these new agents, as noted in some reports, underscores the relentless adaptability of microbes.

Beyond traditional antibiotics, this volume explores innovative approaches. The role of bacteriophage therapy, a long-considered but now increasingly viable option for desperate cases with untreatable infections. Similarly, the use of monoclonal antibodies and immunomodulatory agents to augment the host's immune response against specific pathogens or their toxins represents a promising adjunctive strategy, moving therapy beyond direct microbiocidal action to a more holistic support of the infected host.

## Optimizing therapy: antibiotic concentration monitoring and model-informed precision dosing

Recognizing that simply prescribing an appropriate antibiotic is insufficient in the critically ill, a significant portion of this Research Topic is devoted to the pharmacokinetic (PK) challenges unique to this population. The pathophysiological alterations in sepsis and critical illness-such as augmented renal clearance, capillary leak, organ dysfunction, and the use of extracorporeal circuits-lead to highly variable and unpredictable antibiotic concentrations (6). This can result in subtherapeutic exposure (leading to treatment failure and resistance emergence) or supratherapeutic levels (causing toxicity).

Therapeutic drug monitoring (TDM) of antibiotics, particularly for agents with a narrow therapeutic index like vancomycin and aminoglycosides, has been a standard practice. However, some studies advocated for its expansion to β-lactams and other time-dependent antibiotics. Because the results clearly showed that a substantial proportion of critically ill patients fail to achieve target pharmacokinetic/pharmacodynamic (PK/PD) indices for β-lactams with standard dosing, and that TDM-guided dose adjustment improves clinical outcomes (7–10).

This concept is elegantly advanced by the principles of model-informed precision dosing (MIPD). MIPD utilizes population PK models and Bayesian algorithms to individualize dosing regimens from the outset, rather than relying on reactive TDM adjustments. The integration of MIPD into clinical practice represents a monumental leap toward truly personalized antimicrobial therapy, ensuring that each patient receives the right drug at the right dose and the right time.

## The digital frontier: the transformative role of artificial intelligence

Perhaps the most futuristic yet rapidly materializing theme in this Research Topic is the application of Artificial Intelligence (AI) and machine learning (ML) in the ICU. The vast, multidimensional data generated at the bedside-from electronic health records and laboratory results to continuous vital sign monitoring and medical imaging-creates an ideal environment for AI algorithms to identify patterns invisible to the human eye (11–13).

The articles in this section explore several groundbreaking applications. AI models are being developed to predict the risk of sepsis onset hours before clinical recognition, allowing for pre-emptive intervention. Other studies utilize ML to differentiate between colonization and true infection, a classic diagnostic dilemma in the ICU, or to predict the probability of MDRO etiology based on patient demographics, comorbidities, and recent healthcare exposure.

Furthermore, AI is being harnessed to support antimicrobial stewardship. Algorithms can analyze local epidemiology and patient-specific data to recommend optimal empiric antibiotic regimens or flag patients for automatic infectious diseases consultation. The potential for AI to integrate real-time data on pathogen identification (from rapid diagnostics), antibiotic levels (from TDM), and clinical response to provide dynamic, patient-specific treatment recommendations is no longer science fiction but an active area of research, as highlighted in this volume.

## Deconstructing heterogeneity: the promise of omics-defined subphenotypes

A profound realization in critical care medicine is that syndromes like sepsis and acute respiratory distress syndrome (ARDS) are not single diseases but represent a collection of distinct biological subphenotypes with different underlying drivers, clinical trajectories, and responses to therapy. The “one-size-fits-all” approach to clinical trials has largely failed, and the future lies in targeted therapy for specific subpopulations.

This Research Topic showcases the power of “omics” technologies-transcriptomics, proteomics, metabolomics-to uncover these subphenotypes. By analyzing the entire set of genes, proteins, or metabolites in a patient's blood, researchers can identify unique molecular signatures. For instance, some studies have identified two distinct subphenotypes of sepsis: one characterized by a hyperinflammatory response and another by immunosuppression and T-cell exhaustion. These subphenotypes may benefit from diametrically opposed therapies-immunosuppression vs. immunostimulation-explaining the failure of previous generalized immunomodulatory trials.

The clinical application of this knowledge is evolving from complex laboratory assays toward point-of-care classifiers (14). Studies here explore the use of limited protein panels or even electronic health record data that can accurately assign patients to a specific subphenotype rapidly, enabling future enrichment strategies for clinical trials and, ultimately, personalized administration of biologics and immunomodulators.

## Conclusion: toward a synergistic and personalized future

The 22 articles compiled in “*Infections in the Intensive Care Unit - Volume III*” collectively chart a course for the future of our field. That future is one where the grim reality of AMR is met not with despair but with a coordinated, technologically sophisticated counteroffensive. It is a future where rapid diagnostics illuminate the pathogen within hours, where novel antimicrobials and adjunctive therapies provide effective treatment options, and where MIPD ensures these powerful drugs are used with precision.

Most excitingly, it is a future where AI acts as a powerful co-pilot, synthesizing vast datasets to predict, diagnose, and recommend, and where the biological heterogeneity of our patients is decoded through omics, allowing us to move from treating a syndromic label to treating a specific, molecularly-defined disease process.

The journey ahead requires collaboration among intensivists, microbiologists, clinical pharmacists, data scientists, and translational researchers. The work presented in this Research Topic is a testament to the vibrant and innovative spirit of this community. By embracing and integrating these advancements, we can transform the ICU from a battleground where we are often outmaneuvered by microbes into a center of precision medicine, where every infected critically ill patient receives the uniquely tailored care they deserve.
